# Patient’s punctuality in an outpatient clinic: the role of age, medical branch and geographical factors

**DOI:** 10.1186/s12913-023-10379-w

**Published:** 2023-12-11

**Authors:** Benedetta Cerruti, Davide Garavaldi, Alberto Lerario

**Affiliations:** Santagostino, Via Temperanza 6, Milano, 20127 Italy

**Keywords:** Outpatient clinic, Punctuality, Clinical management, Age

## Abstract

**Background:**

The efficiency of the management of an outpatient clinic largely depends on the administration of patient flows and waiting times increase costs and affect clinical quality. In this study, we verify if the visit acceptance times are influenced by demographic or geographical factors in a large cohort of patients referred to a city and suburban private outpatient multidisciplinary clinic.

**Methods:**

We included all scheduled visits of patients aged from 18 to 75 years who arrived in 2021, 2022 and 2023 in our private outpatient clinics, consisting of 34 medical clinics scattered in Milan metropolitan city and hinterland. The variables collected were age, visit time, check-in time, address of the medical clinic and its distance from the closest underground station, patient typology (new business vs. follow-up patient), and the medical branch of the visit. Outcome is’punctuality’, defined as check-in time minus visit time (in minutes).

**Results:**

We considered a sample of 410.808 visits from January 2021 to April 2023. The majority of patients check-in early (84.4%) and we found that the percentage of punctual patients increases linearly with age. Earlier hours in the morning show the worst punctuality pattern as well as Blood Draws in the analysis of different medical branches. We also observed that patients who already had some activity recorded in our systems show the worst pattern of punctuality. No particular differences emerged considering the geographical location of the clinics.

**Conclusions:**

Younger patients have worse punctuality than older patients. Moreover, earlier hour slots are the most disadvantaged and the medical specialty has an influence on the arrival habits. This data should be considered for better clinical quality and efficiency.

**Supplementary Information:**

The online version contains supplementary material available at 10.1186/s12913-023-10379-w.

## Background

Optimizing times is essential to improve the service of outpatient clinics for physicians and patients. Patient's unpunctuality makes it difficult to manage agendas and increases costs [1–3], prolongs clinical sessions [4, 5], significantly influencing the perceived patient’s satisfaction: the longer those patients wait, the lower their reported satisfaction levels are. Of course, the organization of the physicians also affects waiting time, but there are few studies on this topic. Bleustein et al. highlight how patients, on average, can spend up to twenty minutes in the waiting room due to a doctor's delay [6]. It is also important to underline how punctuality varies during the day as well: as emerges from the literature, patients tend to arrive earlier as the day goes by, being less punctual in the early morning and more punctual in the late evening [7].

Both a delay and an excessive advance with respect to the visiting time can create logistical problems in patient flow management and clinical quality [8–10]. Excessive time advance may not be desirable, as it can cause unwanted congestion of waiting areas with consequent difficulties in managing patients, especially considering the regulations following the COVID-19 pandemic. While regarding reduced punctuality, some data in neurological outpatients’ clinics suggest that the younger the people are, the more they tend to arrive in the clinic later than the fixed time, while older people arrive earlier [11].

Some analytical simulations, carried out to evaluate how to reduce the delay, have revealed that acceptance systems and clinical services that strictly adhere to pre-established timetables lead to a reduction of delays of up to 5% of the average timetable [9].

The analysis carried out so far concerns small samples of patients affected by specific diseases in different contexts. Therefore, the aim of this study is to verify if the visit acceptance times are influenced by demographic or geographical factors in a large cohort of patients referred to a city and suburban private outpatient clinic where all medical specialties are present and both chronic and acute pathologies are treated.

## Methods

### Study population and setting

This study is a retrospective cohort study performed at our private outpatient clinics consisting of 34 medical clinics scattered in Milan metropolitan city and hinterland. All the city clinics can be reached by public transport, and the out-of-town locations by car, with nearby parking garages.

We included all scheduled appointments of patients aged from 18 to 75 years who arrived in our clinics in 2021, 2022 and 2023, that is when the period of hardest COVID-19 emergency had passed and flows 'at full capacity' can reasonably be assumed. Average age of our sample is (46,5 ± 13,9) years, 40% males and 60% females, in line with our habitual patient base (for more information about the population of the analyzed sample, see the supplementary material, Table S1). The patients’ range of ages was decided on the basis of their presumably motor and movement autonomy [12], so that the punctuality of the visit reasonably depended only on the patient and not on any accompanying person. For underage children and people over 75 years old, in fact, their punctuality may not be reflecting their own behavior. All data was collected by the front-desk staff on arrival or by automated procedures. The study had the only exclusion criteria of psychological examinations due to heterogeneity in check-in procedures with respect to our other medical branches. In our clinics there are digitized or in-person booking, acceptance and payment systems, and we recommend maximum punctuality even using the day before and the same day reminders via SMS and email. We calculated the frequency of late check-ins for patient age and medical branch, as the ratio between the number of late check-ins and the total number of check-ins.

In our clinics we perform medical examinations of all clinical specialties and minor surgery for acute, episodic and chronic diseases, blood sampling, diagnostic imaging, dentistry, physiotherapy and psychological examinations (the only exclusion). Patients attending the outpatient clinics are either new referrals referred by their general practitioner (GP), other medical specialists or on their own initiative or follow-up patients.

### Outcomes

The variables collected were age, appointment time, check-in time, address of the medical clinic, patient typology (new business vs. follow-up patient), and the medical branch of the appointment. Outcome is’punctuality’, defined as check-in time minus appointment time (in minutes). Negative values reflect the waiting time for early arrivals and positive (or zero) values are associated with delay time for late arrivals.

### Statistics

Our statistical sample includes appointments from January 2021 to April 2023, to avoid COVID-19 pandemic effects on our regular activity due to Italian government restrictions for spreading limitations, or to any other possible related effect (direct or indirect). The sample includes 410.808 visits (see Table S1 for more information).

**Fig. 1:**
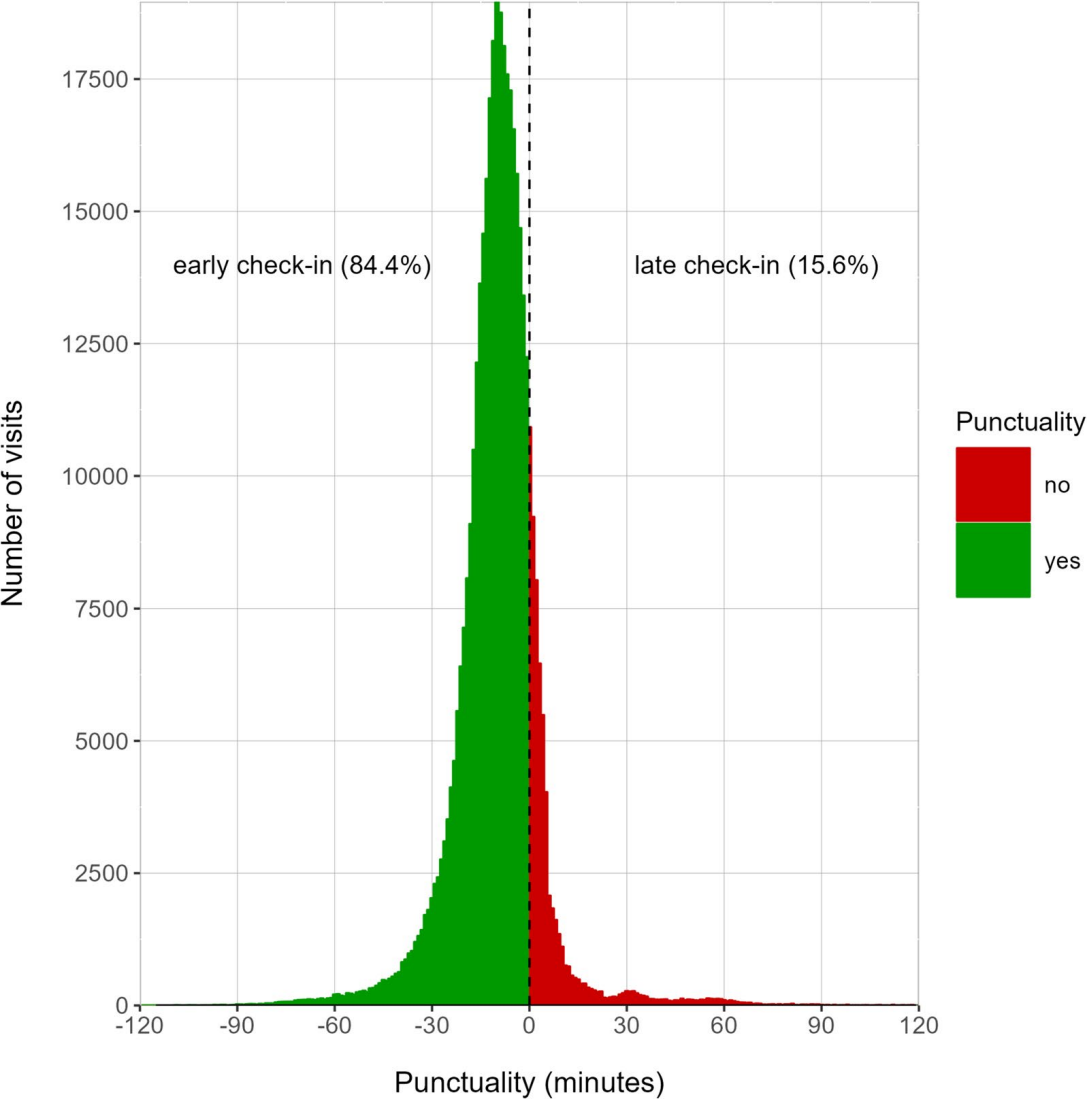
Distribution of punctuality for our patients, years 2021–2023 (green for punctual, red for delay). Punctuality is defined as the check-in time minus the visit time (in minutes). The majority of patients (84,4%) check-in before the scheduled time for the visit. The distribution is not Normal, according to Q-Q plot and Kolmogorov–Smirnov test (see supplementary material for detail, Fig. S1)

In order to study the normality of the distribution of punctuality (Fig. 1), both Kolmogorov–Smirnov test and Q-Q plot were performed. For detail, see the supplementary material (Figure S1).

Linear regression was used to study the relation between patients’ age and the average punctuality. Coefficient of determination R^2^ and the Pearson correlation coefficient ρ were calculated, in order to evaluate the linearity of the model and the correlation strength.

## Results

We considered a sample of 410.808 visits from January 2021 to April 2023. Check-in process may be either automatic when self-check-in is used by the patient, or performed by front-end staff. Both procedures record the timestamp of the check-in in our systems. Punctuality is defined as the difference between the check-in time and the time scheduled for the appointment of the patient. Hence, according to our operative definition, negative values correspond to patients who checked-in early, e.g. prior to the appointment time, while positive (or zero) values are associated with delays, as commonly defined in literature. The majority of patients check-in early (84.4%), with mode -10 min (e.g. 10 min prior to visit time), Fig. 1. Punctuality does not follow a Normal distribution (see supplementary materials for detail, Figure S1).

We thus analyzed the association of punctuality with various features, namely age, visit scheduled time, medical branch of the service, and geographical detail of the location (locations within 1 km distance from the closest underground station, that we call Group 1, or farther, namely Group 2, either inside metropolitan city of Milan, hinterland or small towns). We can also estimate the frequency of punctuality for our features, by normalizing the punctuality events for the total number of events for a given value of the considered feature.

**Fig. 2:**
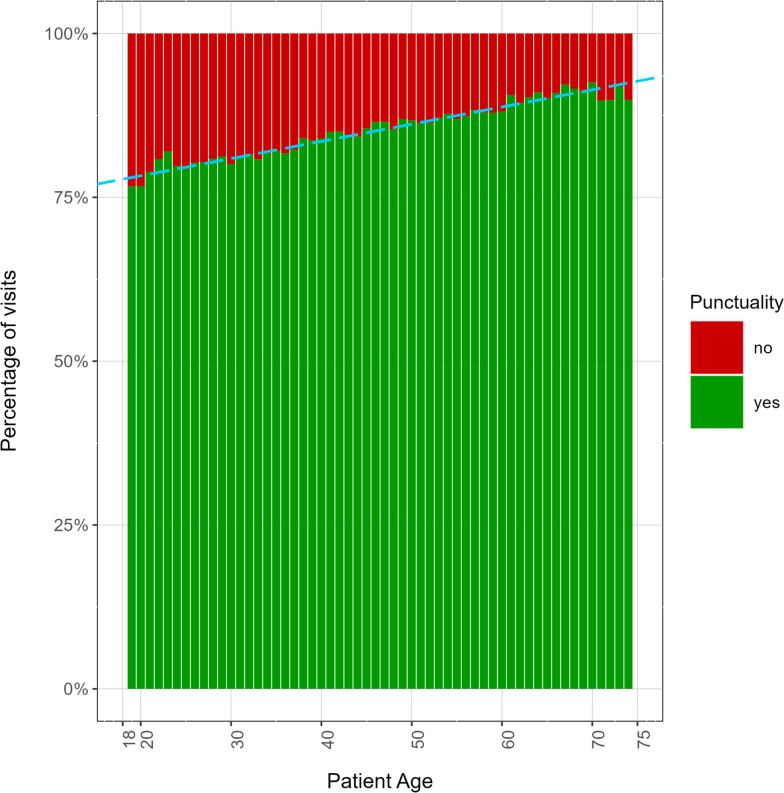
Percentage of punctual visits as a function of the patient’s age (green for punctual, red for delay). Patient’s age is expressed in years. The percentage of punctual patients increases linearly with age. (Linear fit: R.^2^ = 0.92; high level of significance: *p*-value < 0.001; Pearson correlation: *ρ* = -0.96)

The percentage of delay in medical appointments shows a linear association with the patient’s age (R^2^ = 0.92, *p*-value < 0.001, Pearson correlation *ρ* = -0.96). We can thus claim that lower patient’s ages are associated to higher percentages of delay (Fig. 2).

**Fig. 3:**
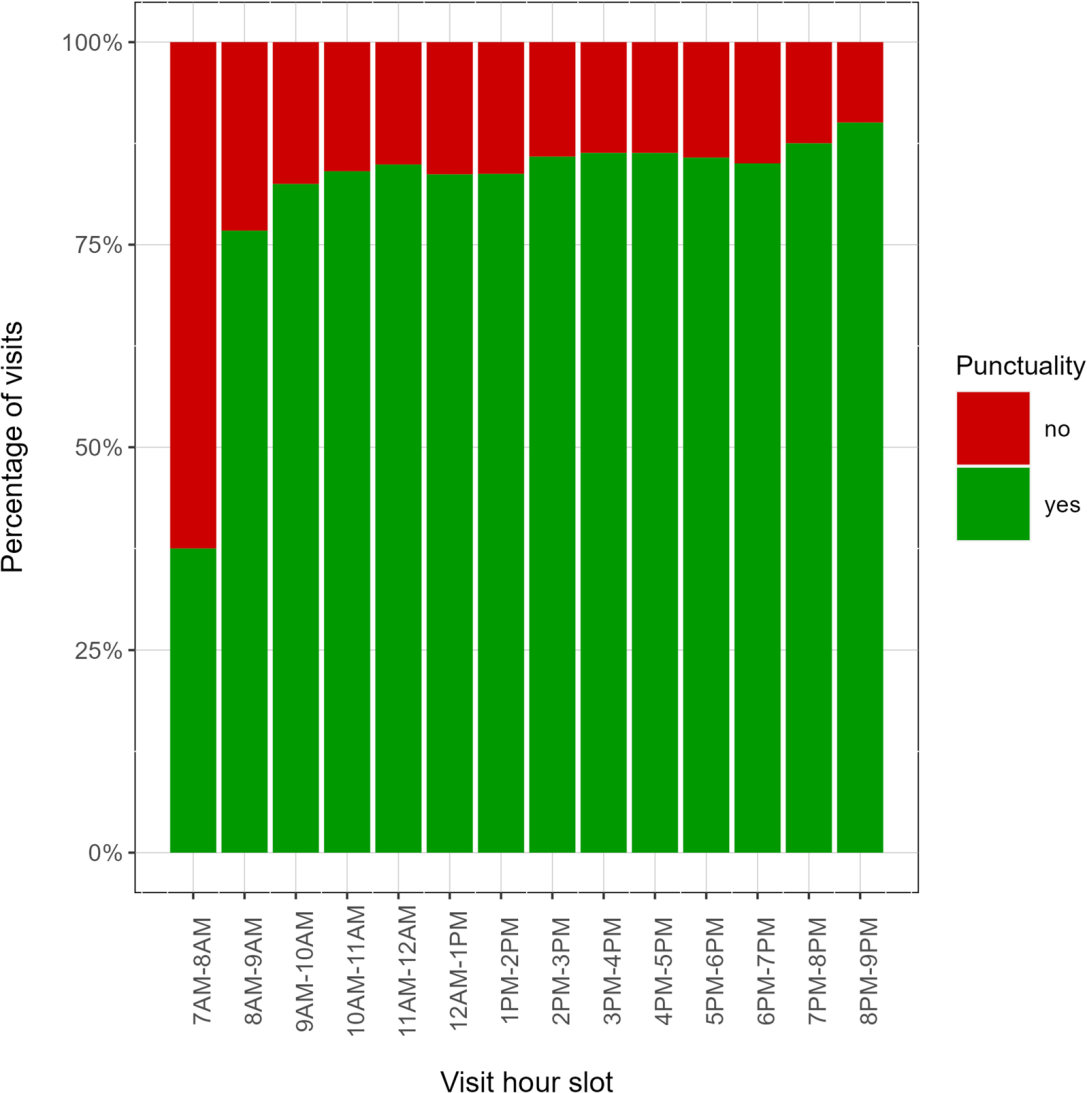
Percentage of punctuality by visit hour slot (green for punctual, red for delay). Early hours in the morning are more likely to be affected by unpunctuality

Patient’s age is not the only factor affecting punctuality: we observed, in agreement with literature, that earlier hours in the morning show the worst punctuality pattern, while late afternoon is the more punctual slot (Fig. 3).

**Fig. 4:**
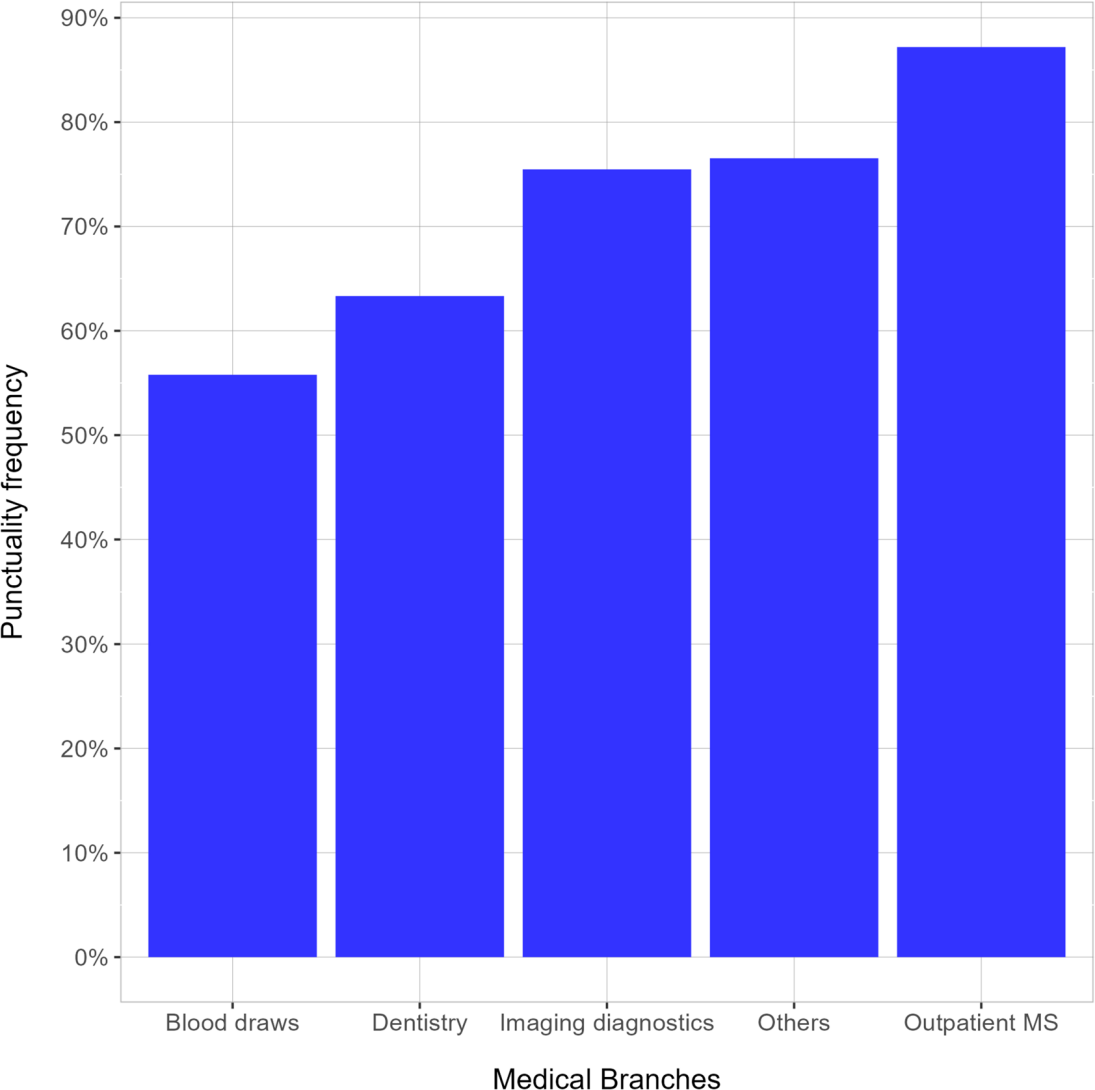
Punctuality frequency for the different medical branches. ‘Blood draws’ is the branch with the worst percentage of patients’ punctuality, about 55% of punctual visits (45% of delays)

Observed delay frequencies are also shown in Fig. 4 for the different medical branches.. ‘Blood draws’ includes all the laboratory specialties, while ‘Dentistry’ spans from orthodontia to oral surgery. ‘Imaging diagnostics’ includes ultrasound, endoscopy, radiography, CT with and without contrast, MRI with and without contrast. ‘Others’ includes vaccines, physiotherapy, osteopathy, orthoptics, obstetrics, physical therapies. ‘Outpatient Medical and Surgery (MS)’ includes all other medical specialties. It is important to notice that ‘Blood draws’, the branch showing the worst punctuality pattern, has a prevalence of visits in the earlier morning slots, thus affecting its global punctuality.

**Fig. 5:**
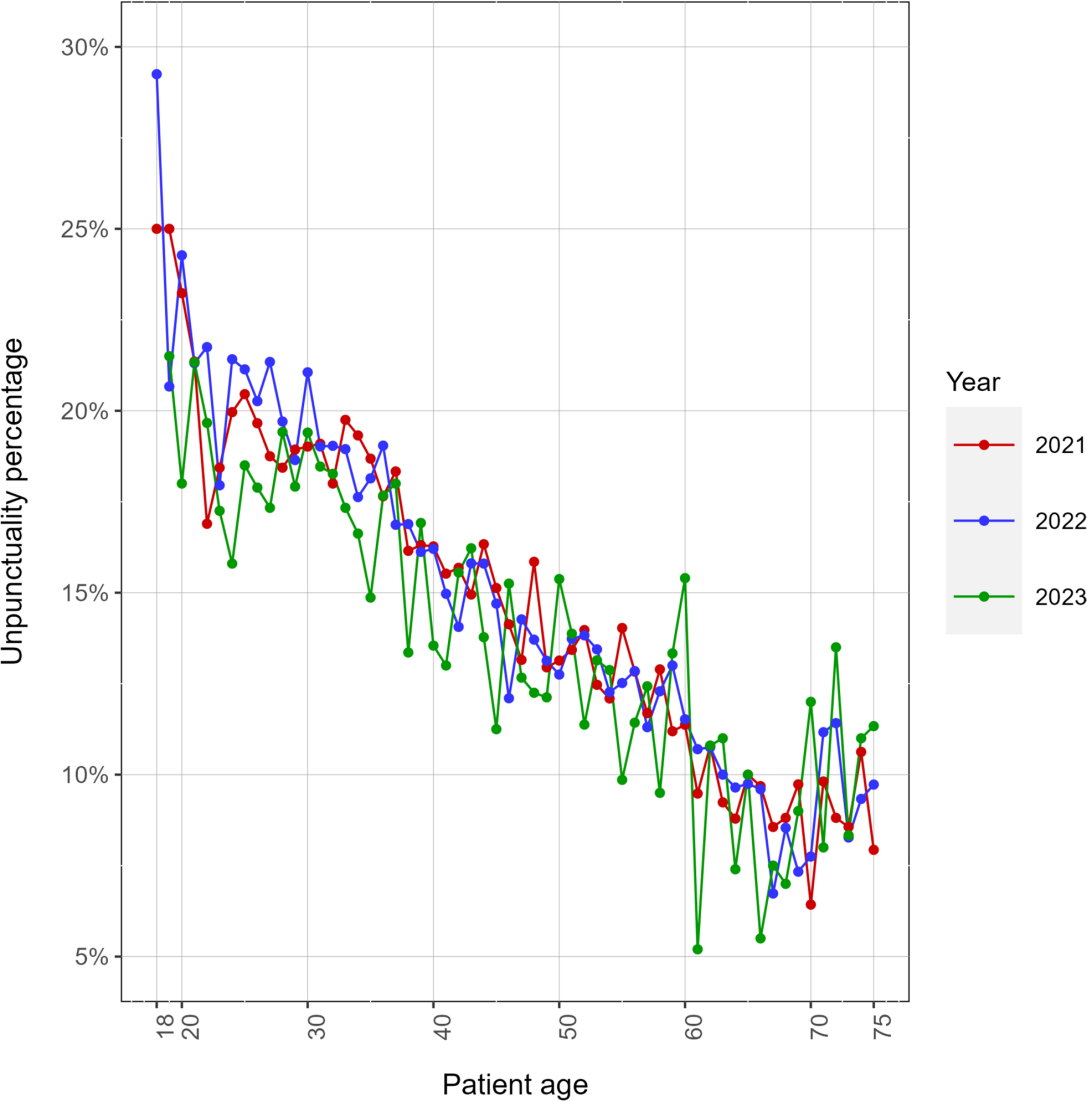
Unpunctuality percentage as a function of the patient’s age by year. The three curves have a similar behavior. 2023’s 18 years old do not appear in the figure, due to the low statistics

It is also interesting to notice that the patient’s punctuality pattern with age does not qualitatively vary in the three different years analyzed in Fig. 5. This means that the pattern is somehow specific to the age of the patient, which is a non-trivial result, especially considering that around 40% of our patients come back the following year (data not shown).

**Fig. 6:**
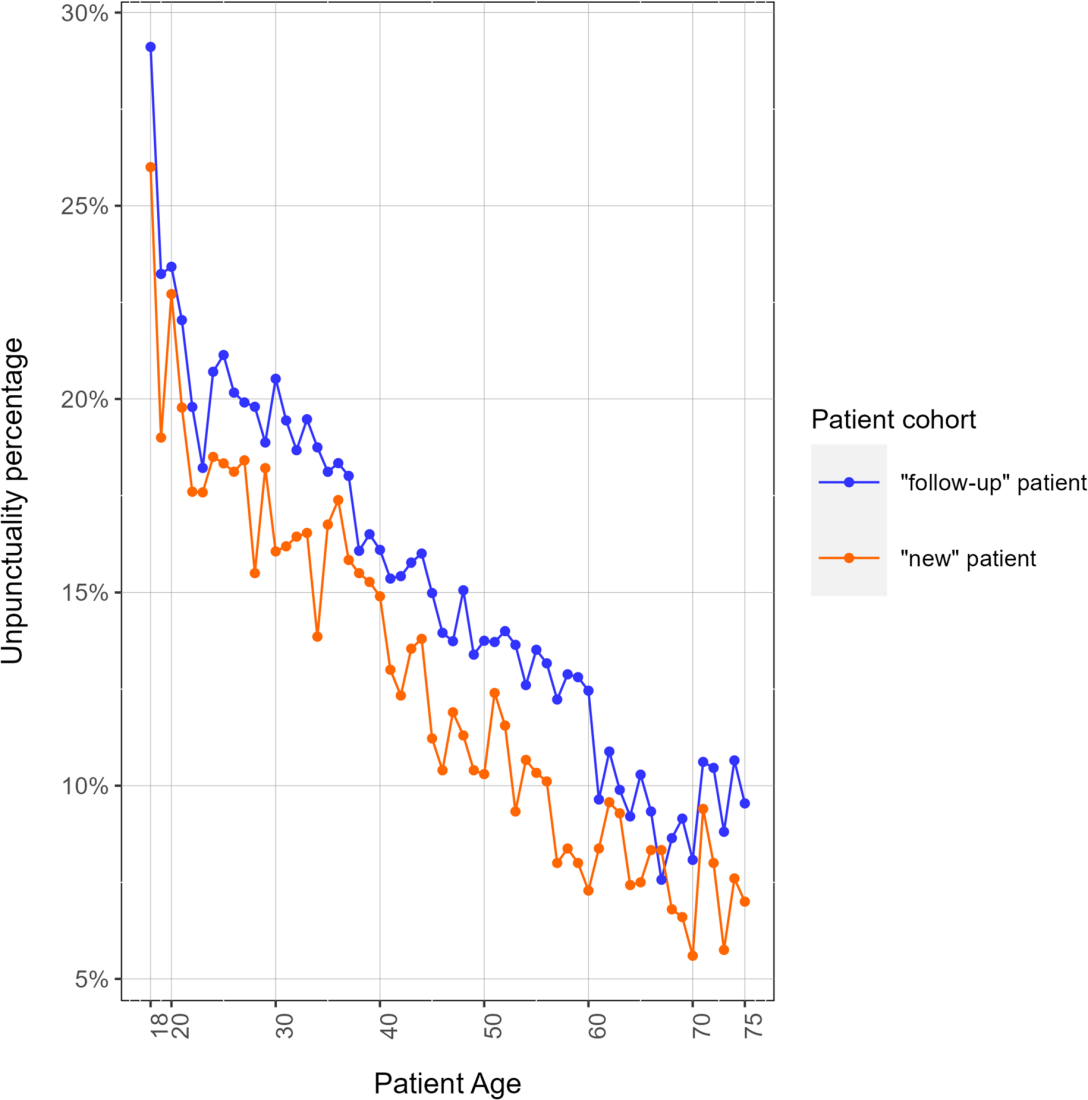
Unpunctuality percentage as a function of the patient age (in years) by different patient cohorts: “new” patient (orange) is a person that has never come before to any of our medical centers, while “follow-up” patients (blue) already have some activity recorded in our systems. “Follow-up” patients show a worse punctuality pattern than “new” patients

Next, we analyzed the frequency of punctuality events for “follow-up patients” vs “new patients”, where we defined a “new” patient as a person that had never come before to any of our medical centers, while “follow-up” patient as a person that had already had some activity recorded in our systems, including the ones in different medical branches. “Follow-up” patients show a shifted (worst) pattern of punctuality (Fig. 6). Further analysis on the topic would be useful and will be addressed in the future, since a larger sample would allow to better investigate the significance of the shift.

Finally, we analyzed the pattern of punctuality as a function of age separately for our locations within 1 km from the closest underground station (23 locations, Group 1) and the others (11 locations, Group 2). No qualitative difference was found for the two cohorts (Figure S2).

## Discussion

In this contribution we aim at studying the associations between trends in punctuality at check-in for medical appointments and several features related with our patients, namely age, category of appointment, appointment hour slot and the position of our locations with respect to the closest public transport. To the best of our knowledge, this is the largest multidisciplinary sample (410.808 visits) ever considered in similar studies.

Previous studies show a range of 5% to 10% of unpunctual patients [9, 11, 13], in this study 84,4% of patients showed up early and 15,6% late, mainly concentrated between 15 min before or after the scheduled appointment. However, a large percentage of patients arrive earlier or later than 15 min for their appointments. This degree of unpunctuality increases the risk of overcrowded waiting areas, the physician’s distress and reduces patient satisfaction and clinical performance.

As previously pointed out in literature, we confirm that younger patients have a stronger tendency to delays [11]. We found that the younger the patient, the (linearly) larger the percentage of late check-ins and the amount of the delay. Interestingly, we noticed the same type of pattern for all the observed years.

The frequency of delays is also affected by the appointment hour slot: early appointments in the morning are more prone to late check-ins, while the late afternoon is the more punctual slot, in qualitative agreement with previous findings [7]. Moreover, the vast majority of the appointments of the slot 7AM-8AM belong to the Blood Draws branch, which in fact displays the larger probability of late check-in. On the other hand, Imaging Diagnostic, Physical Therapies and Medical-Surgery appointments show a progressively higher percentage of punctuality. Age-related earliness could be related to habits and attitudes: the awareness of one's own health and consequences of the impact of a disease varies according to age [14]; young people tend to go to sleep later and struggle to get up early in the morning [15, 16]; the reason for a medical appointment also matters, in fact in our analysis routinary blood tests show less punctuality. As also reported in the literature, blood tests can probably be underestimated [17] as they are not considered strictly related to an immediate clinical response.

The difference between "follow-up patients" and "new patients" is not statistically evident, even if our data show a worst punctuality trend in the “follow-up” patients’ cohort. Thus, further investigation is needed to evaluate in our use case our punctuality "recommendation" systems, which are considered effective in the literature: digitized or in-person booking, acceptance and payment systems [18], day before and same day appointment reminder via SMS and email [19].

Punctuality is usually also associated with various logistical factors (public transport, roads, etc.). However, in our study no particular differences emerged considering the geographical location of the clinics, so the ease or otherwise of reaching a clinic does not seem a matter of fundamental importance, at least inside the Milan area.

Unpunctuality is an important issue in management of outpatient clinics with large implications for patients and physicians. Our clinic, while following the procedures recommended in the literature on appointment management, and while using recommended procedures, has to deal with the non-punctuality of patients which still appears to be consistent. Probably the most useful intervention remains “the inflexibility” on respecting schedules, as reported by Williams et al. in a pain clinic in Baltimore, USA, in which patients were informed that late patients would not be seen and would be rescheduled [9]. Future managerial interventions should consider a subdivision of the scheduled appointment according to the demographic data indicated, associating them with the strictest recommendations on compliance with punctuality.

Some limitations need to be addressed. Respect for timetables is an important social behavior that varies substantially across countries and across individuals [20–23], this study is based on data collected from private outpatient clinics predominantly located in a northern Italian urban area. Therefore, although the cohort is large and multidisciplinary, the type of patients and their needs cannot be generalized for all contexts.

## Conclusions

Patients of our private outpatient clinics check-in mainly earlier than scheduled times for their visits. Younger patients show the worst delay habits; earlier hour slots in the morning are the most disadvantaged and the medical specialty has an influence on the arrival habits. This habit is the same for all the observed years. Further analyses are necessary to establish if in other regions of Italy or in other countries the same pattern is shown. No evident association is found between the punctuality habits and the location of the medical center.

These findings may have actual implications on resource planning in order to maximize the efficiency and improve the patient experience in our outpatient clinics.

### Supplementary Information

**Additional file 1:** **Figure S1.** Normal Q-Q plot of punctuality distribution. Data are not normally distributed since there is a large difference between cumulative Normal distribution and the cumulative distribution of our variable. The result is also confirmed by the Kolmogorov-Smirnov test which rejects the hypothesis of normality (*p*-value < 2.2e-16). **Figure S2.** Unpunctuality percentage as a function of the patient's age for different types of medical location: ‘Group 1’ includes locations within 1 km from the closest underground station (23 locations, blue) and the others correspond to ‘Group 2’ (11 locations, orange). The two groups do not show a qualitative difference. **Table S1.** Cohort characteristics by relevant patients’ features. For each feature the total number of visits is shown; this number is then split into two other columns, the first one with the number of visits where the patient checked-in late and the second one with the number of visits where the patient checked-in early.

## Data Availability

The datasets used and/or analyzed during the current study are available from the corresponding author on reasonable request.

## Abbreviations

GP: General practitioner
SMS: Short message service
CT: Computed tomography
MRI: Magnetic resonance imaging
