# Supplementary material for: Patient’s punctuality in an outpatient clinic: the role of age, medical branch and geographical factors
Source: BMC Health Serv Res. 2023 Dec 11;23:1385. doi: 10.1186/s12913-023-10379-w (PMC10714636; doi:10.1186/s12913-023-10379-w)
Supplement: Supplementary file 1 — Additional file 1: Figure S1. Normal Q-Q plot of punctuality distribution. Data are not normally distributed since there is a large difference between cumulative Normal distribution and the cumulative distribution of our variable. The result is also confirmed by the Kolmogorov-Smirnov test which rejects the hypothesis of normality (p-value < 2.2e-16). Figure S2. Unpunctuality percentage as a function of the patient's age for different types of medical location: ‘Group 1’ includes locations within 1 km from the closest underground station (23 locations, blue) and the others correspond to ‘Group 2’ (11 locations, orange). The two groups do not show a qualitative difference. Table S1. Cohort characteristics by relevant patients’ features. For each feature the total number of visits is shown; this number is then split into two other columns, the first one with the number of visits where the patient checked-in late and the second one with the number of visits where the patient checked-in early. [file 12913_2023_10379_MOESM1_ESM.docx]

**Supplementary material**

**Distribution of punctuality**

In order to study the normality of the distribution of punctuality (Fig.1), both Kolmogorov-Smirnov test and Q-Q plot (Fig.S1) were performed. The first one is used to compare the distribution of a sample with a reference distribution (Normal distribution for the present case) and the second one is used to compare the cumulative distribution of the observed variable with the cumulative Normal distribution. Due to the p-value of the Kolmogorov-Smirnov test, the hypothesis of normality of the distribution was rejected (p-value < 2.2e-16). The Q-Q plot confirmed the non-normality of the data, since cumulative distribution of our variable was very different from the cumulative Normal distribution.


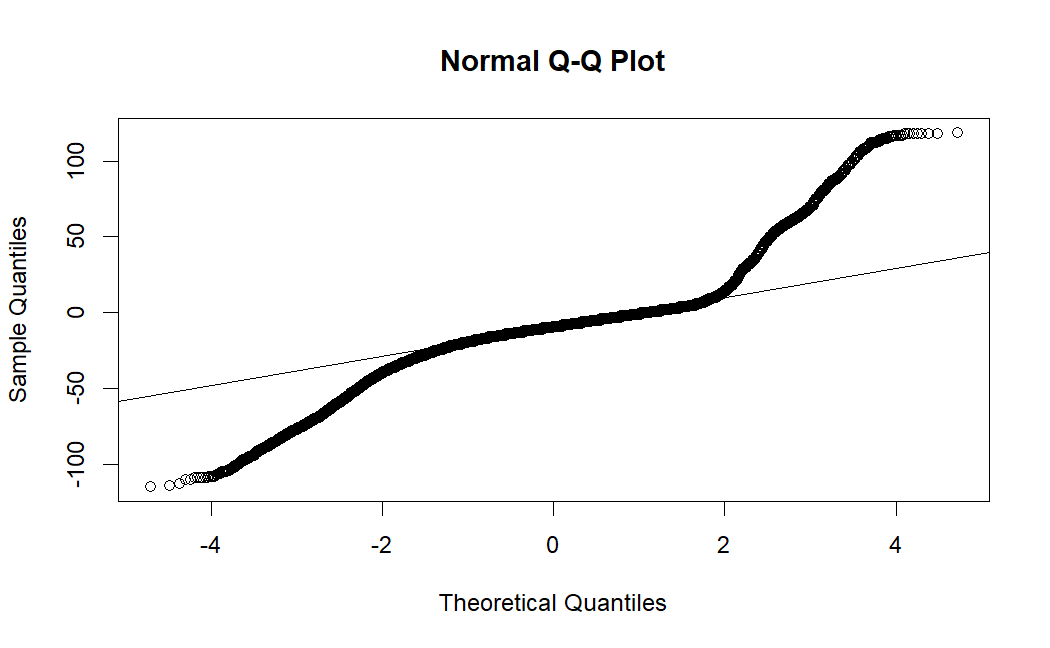


**Figure S1**: Normal Q-Q plot of punctuality distribution. Data are not normally distributed since there is a large difference between cumulative Normal distribution and the cumulative distribution of our variable. The result is also confirmed by the Kolmogorov-Smirnov test which rejects the hypothesis of normality (p-value < 2.2e-16).

**Unpunctuality distribution for different ages and geographical location of the clinics**


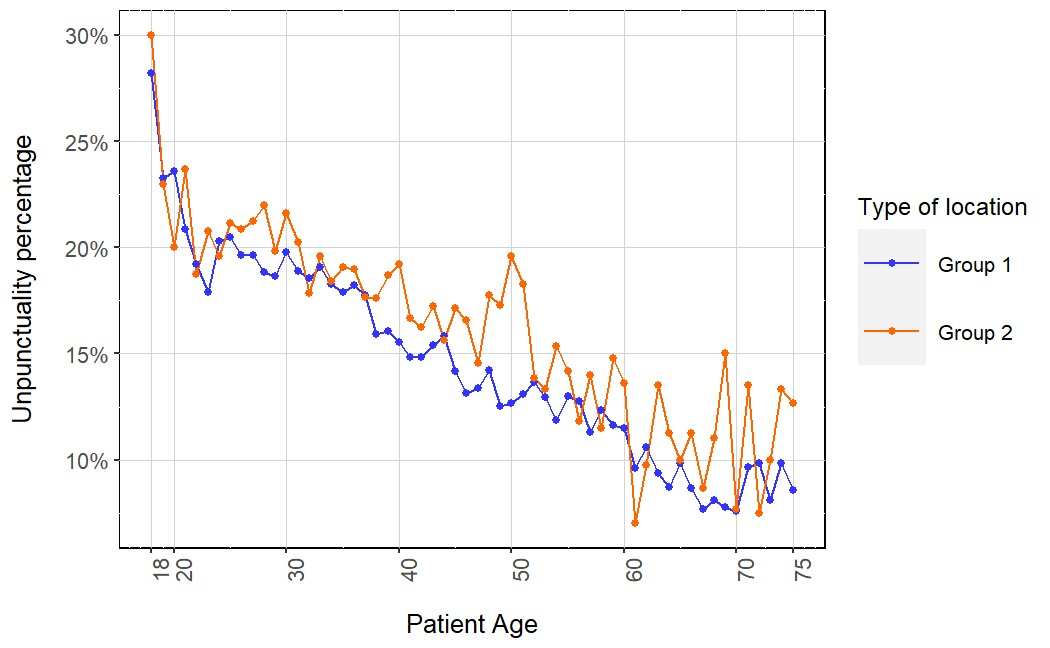


**Figure S2:** Unpunctuality percentage as a function of the patient's age for different types of medical location: ‘Group 1’ includes locations within 1 km from the closest underground station (23 locations, blue) and the others correspond to ‘Group 2’ (11 locations, orange). The two groups do not show a qualitative difference.

**Visits characterization for relevant patients’ features**

|  | **Overall cohort** | **Check-in late** | **Check-in early** |
| --- | --- | --- | --- |
| Age - 18 | 1212 | 341 | 871 |
| Age - 19 | 2337 | 534 | 1803 |
| Age - 20 | 2760 | 641 | 2119 |
| Age - 21 | 3446 | 729 | 2717 |
| Age - 22 | 3964 | 760 | 3204 |
| Age - 23 | 4965 | 897 | 4068 |
| Age - 24 | 5709 | 1157 | 4552 |
| Age - 25 | 7314 | 1504 | 5810 |
| Age - 26 | 8494 | 1677 | 6817 |
| Age - 27 | 9651 | 1899 | 7752 |
| Age - 28 | 11252 | 2149 | 9103 |
| Age - 29 | 12230 | 2293 | 9937 |
| Age - 30 | 13194 | 2633 | 10561 |
| Age - 31 | 13436 | 2550 | 10886 |
| Age - 32 | 14213 | 2621 | 11592 |
| Age - 33 | 13721 | 2629 | 11092 |
| Age - 34 | 13394 | 2446 | 10948 |
| Age - 35 | 12553 | 2257 | 10296 |
| Age - 36 | 11942 | 2178 | 9764 |
| Age - 37 | 11707 | 2077 | 9630 |
| Age - 38 | 11211 | 1797 | 9414 |
| Age - 39 | 10674 | 1742 | 8932 |
| Age - 40 | 9534 | 1520 | 8014 |
| Age - 41 | 8725 | 1311 | 7414 |
| Age - 42 | 8113 | 1215 | 6898 |
| Age - 43 | 8117 | 1255 | 6862 |
| Age - 44 | 7959 | 1255 | 6704 |
| Age - 45 | 7867 | 1140 | 6727 |
| Age - 46 | 7848 | 1061 | 6787 |
| Age - 47 | 7965 | 1074 | 6891 |
| Age - 48 | 8051 | 1174 | 6877 |
| Age - 49 | 8038 | 1042 | 6996 |
| Age - 50 | 7512 | 997 | 6515 |
| Age - 51 | 7422 | 1007 | 6415 |
| Age - 52 | 7607 | 1038 | 6569 |
| Age - 53 | 6827 | 889 | 5938 |
| Age - 54 | 6779 | 831 | 5948 |
| Age - 55 | 6770 | 887 | 5883 |
| Age - 56 | 6358 | 805 | 5553 |
| Age - 57 | 6121 | 714 | 5407 |
| Age - 58 | 5940 | 728 | 5212 |
| Age - 59 | 5559 | 668 | 4891 |
| Age - 60 | 5595 | 658 | 4937 |
| Age - 61 | 5127 | 483 | 4644 |
| Age - 62 | 5035 | 538 | 4497 |
| Age - 63 | 4477 | 438 | 4039 |
| Age - 64 | 4730 | 423 | 4307 |
| Age - 65 | 4251 | 418 | 3833 |
| Age - 66 | 4001 | 365 | 3636 |
| Age - 67 | 3639 | 282 | 3357 |
| Age - 68 | 3444 | 288 | 3156 |
| Age - 69 | 3244 | 285 | 2959 |
| Age - 70 | 3084 | 233 | 2851 |
| Age - 71 | 3222 | 331 | 2891 |
| Age - 72 | 3155 | 317 | 2838 |
| Age - 73 | 3095 | 259 | 2836 |
| Age - 74 | 3184 | 323 | 2861 |
| Age - 75 | 3034 | 272 | 2762 |
| 7AM-8AM | 706 | 448 | 258 |
| 8AM-9AM | 22230 | 5172 | 17058 |
| 9AM-10AM | 45373 | 7945 | 37428 |
| 10AM-11AM | 47269 | 7532 | 39737 |
| 11AM-12AM | 45094 | 6815 | 38279 |
| 12AM-1PM | 32482 | 5297 | 27185 |
| 1PM-2PM | 20529 | 3333 | 17196 |
| 2PM-3PM | 32506 | 4598 | 27908 |
| 3PM-4PM | 37322 | 5107 | 32215 |
| 4PM-5PM | 36855 | 5038 | 31817 |
| 5PM-6PM | 36599 | 5221 | 31378 |
| 6PM-7PM | 33861 | 5069 | 28792 |
| 7PM-8PM | 18303 | 2287 | 16016 |
| 8PM-9PM | 1679 | 173 | 1506 |
| Blood draws | 9135 | 4039 | 5096 |
| Dentistry | 1399 | 513 | 886 |
| Imaging diagnostics | 3062 | 751 | 2311 |
| Others | 73880 | 17334 | 56546 |
| Outpatient MS | 323332 | 41398 | 281934 |
| Year 2021 | 200935 | 169516 | 31419 |
| Year 2022 | 163272 | 137464 | 25808 |
| Year 2023 | 46601 | 39793 | 6808 |
| "follow-up" patient | 351953 | 56030 | 295923 |
| "new" patient | 58855 | 8005 | 50850 |

**Table S1**: Cohort characteristics by relevant patients’ features. For each feature the total number of visits is shown; this number is then split into two other columns, the first one with the number of visits where the patient checked-in late and the second one with the number of visits where the patient checked-in early.
